# Entorrhizomycota: A New Fungal Phylum Reveals New Perspectives on the Evolution of Fungi

**DOI:** 10.1371/journal.pone.0128183

**Published:** 2015-07-22

**Authors:** Robert Bauer, Sigisfredo Garnica, Franz Oberwinkler, Kai Riess, Michael Weiß, Dominik Begerow

**Affiliations:** 1 University of Tübingen, Plant Evolutionary Ecology, Institute of Evolution and Ecology, Auf der Morgenstelle 1, 72076, Tübingen, Germany; 2 University of Tübingen, Department of Biology, Auf der Morgenstelle 1, 72076, Tübingen, Germany; 3 Institute of Organismal Mycology and Microbiology, Vor dem Kreuzberg 17, 72076, Tübingen, Germany; 4 University of Bochum, AG Geobotanik, Universitätsstraße 150, 44780, Bochum, Germany; University of Florida, UNITED STATES

## Abstract

*Entorrhiza* is a small fungal genus comprising 14 species that all cause galls on roots of Cyperaceae and Juncaceae. Although this genus was established 130 years ago, crucial questions on the phylogenetic relationships and biology of this enigmatic taxon are still unanswered. In order to infer a robust hypothesis about the phylogenetic position of *Entorrhiza* and to evaluate evolutionary trends, multiple gene sequences and morphological characteristics of *Entorrhiza* were analyzed and compared with respective findings in Fungi. In our comprehensive five-gene analyses *Entorrhiza* appeared as a highly supported monophyletic lineage representing the sister group to the rest of the Dikarya, a phylogenetic placement that received but moderate maximum likelihood and maximum parsimony bootstrap support. An alternative maximum likelihood tree with the constraint that *Entorrhiza* forms a monophyletic group with Basidiomycota could not be rejected. According to the first phylogenetic hypothesis, the teliospore tetrads of *Entorrhiza* represent the prototype of the dikaryan meiosporangium. The alternative hypothesis is supported by similarities in septal pore structure, cell wall and spindle pole bodies. Based on the isolated phylogenetic position of *Entorrhiza* and its peculiar combination of features related to ultrastructure and reproduction mode, we propose a new phylum Entorrhizomycota, for the genus *Entorrhiza*, which represents an apparently widespread group of inconspicuous fungi.

## Introduction

**Fig 1:**
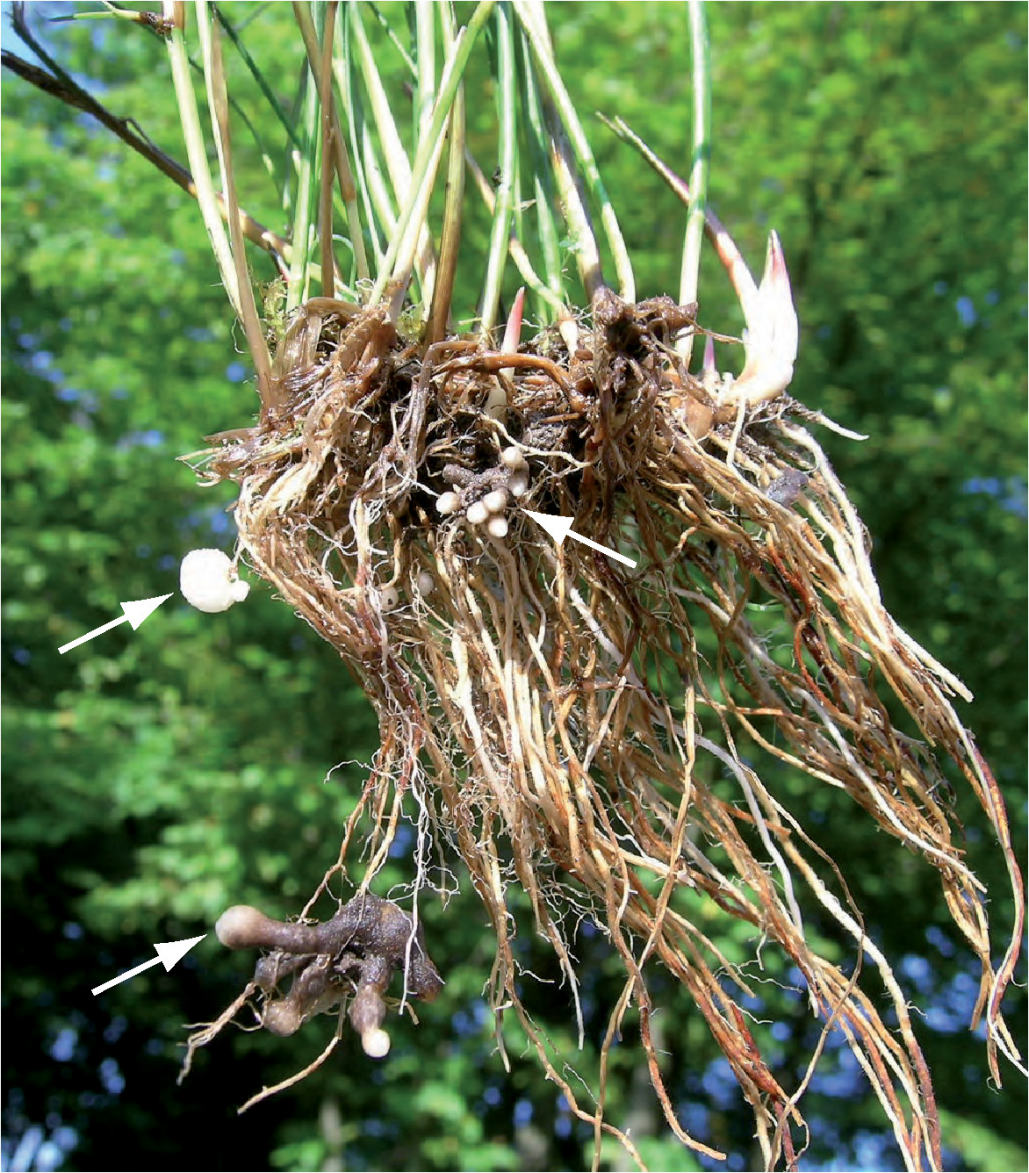
Portion of a tussock of *Juncus articulatus* with root galls caused by *Entorrhiza casparyana* (arrows). Note that the older segments of the galls are brown-colored while younger parts are whitish (tips).

The genus *Entorrhiza* was erected by Weber [1] to describe fungi causing galls on root tips of members of Cyperaceae and Juncaceae (Fig 1). Currently, this small and morphologically uniform genus comprises 14 known species, nine species of which–including the type species *E*. *cypericola*–occur exclusively in roots of members of Cyperaceae (*Carex*, *Cyperus*, *Eleocharis*, *Isolepis*, *Scirpus*), and four occur on members of Juncaceae (*Juncus*). Only *E*. *caricicola* was reported from both Cyperaceae (*Carex*, *Eleocharis*) and Juncaceae (*Juncus*) families [2]. Within the galls *Entorrhiza* forms regularly septate clampless hyphae that are coiled in living host cells, where they terminate with globose cells that detach from the hyphae and become thick-walled teliospores [3–4]. Because of the uniform morphology and the restricted host range it was suggested that this genus represents a monophyletic group [5].

As studied in *Entorrhiza casparyana*, the exosporium of the teliospores is probably formed by the host [6]. The septa have dolipores [4], [7–8]. After a resting period of some months teliospores germinate internally by becoming regularly four-celled “phragmobasidia” [8–10]. Deml and Oberwinkler [4], having used incorrectly labelled preparations for microscopy, illustrated a clamped hypha and a *Tilletia*-like basidium for *E*. *casparyana* [11].

*Entorrhiza* had uncertain positions in recently published phylogenetic studies [12–14]. These studies suggested two possible phylogenetic placements for *Entorrhiza*, either as a sister group of all other Ustilaginomycotina [7–8], [12], [15], or as a sister group of the Basidiomycota [13–14]. However, so far only rDNA genes have been used to infer phylogenetic hypotheses.

In order to achieve a robust estimate for the phylogenetic position of *Entorrhiza* and to evaluate evolutionary trends, we followed two strategies: First, we analyzed nucleotide sequences of the rDNA coding for the 18S, 5.8S and 28S ribosomal subunits together with amino acid sequences of the *rpb*1 and *rpb*2 genes of *E*. *aschersoniana* (on *Juncus bufonius*, Juncaceae), *E*. *casparyana* (on *Juncus articulatus*, Juncaceae) and *E*. *parvula* (on *Eleocharis parvula*, Cyperaceae), together with a representative dataset of the Fungi. Second, we compared morphological characteristics of *Entorrhiza* with homologous structures in other fungal groups.

## Material and Methods

### Ethics statement

The plant and fungal specimen used in this study, as well as the sampling sites, were not protected and therefore no specific permits were requested for sampling.

### Molecular phylogenetic analyses

The following specimens of *Entorrhiza* were used to obtain new sequences (see Table 1): *E*. *aschersoniana* on *Juncus bufonius*, Costa Rica, Prov. Cartago, Irazú, entrance area of the park, 13 Oct 1996, leg. M. Piepenbring, MP2230; *E*. *aschersoniana* on *J*. *bufonius*, Poland, Malopolska Province: Markowa, ca. 60 km SW of Krakow, 49°09'10''N 9°59'48''E, 830 m above sea level (a.s.l.), 8 Sept 2006, leg. J. and M. Piątek, KRAM-56778; *E*. *casparyana* on *J*. *articulatus*, Australia, Tasmania, south of Ben Lomond National Park, road B 42, near Rosarden, 41°37'08.8"S 147°52'07.8"E, 17 March 1996, leg. C. and K. Vánky, HUV 17623; *E*. *casparyana* on *J*. *articulatus*, Germany, Baden-Württemberg, Triensbach, Reusenberg, 49°09'10.3"N 9°59'48.8"E, 2 Oct 2007, leg. R. Bauer, RB 3117; and *E*. *parvula* on *Eleocharis parvula*, France, Gironde, Bassin d’Arcachon, Arès, 44°47'00"N 1°09'02"W, 25 July 2014, leg. M. Lutz and M. Piątek, TUB 021488. For *E*. *casparyana*, we separately amplified *rpb*1 and *rpb*2 from the collections RB 941, RB 3117 and RB 3200, which sequences proved to be identical.

**Table 1:** List of species and GenBank accession numbers used for the combined multiple gene analysis (see Fig 2).

Supraordinal classification	Species	GenBank accession 18S	GenBank accession 5.8S	GenBank accession 28S	GenBank accession RPB1 (A-C)	GenBank accession RPB2
**Dikarya**						
**Ascomycota**						
**Pezizomycotina**						
	*Aspergillus fumigatus*	M55626	KJ716967	AJ438344	XP_752837	AY485610
	*Sclerotinia sclerotiorum*	L37541	GQ375746	DQ470965	EF014371	AF107808
	*Lecanora hybocarpa*	DQ782883	DQ782849	DQ782910	DQ782829	DQ782871
	*Aleuria aurantia*	AY544698	DQ491495	AY544654	DQ471120	DQ247785
	*Neurospora crassa*	X04971	GU327630	AY681158	XP_329293	XP_957106
	*Magnaporthe grisea*	AB026819	KC291448	JX134682	XP_362207	XP_362269
**Taphrinomycotina**						
	*Neolecta vitellina*	NG_013189	KC333874	NG_027573	ABM26988	AF107786
	*Protomyces inouyei*	D11377	DQ497617	AY548294	DQ471135	AY548299
	*Taphrina deformans*	U00971	AF492093	DQ470973	EF014374	AY485633
**Saccharomycotina**						
	*Saccharomyces cerevisiae*	KC969085	KF486912	JN938921	AFF27719	NP_014794
	*Candida albicans*	X53497	EF192231	JN874496	EAL00529	EAK99513
**Basidiomycota**						
**Agaricomycotina**	*Agaricus bisporus*	FJ869172	AM930981	**DQ071710**	**DQ067962**	**FJ623644**
	*Tephrocybe rancida*	**FJ641902**	EU669250	**EU669301**	**FJ623639**	**FJ623645**
	*Hydnum repandum*	AF026641	KC859459	KF294643	EF014376	AY485624
	*Calocera viscosa*	**FJ641897**	DQ520102	DQ520102	**FJ623634**	**FJ623649**
	*Ramaria stricta*	AF026638	AF442097	AF287887	**FJ623637**	**FJ623648**
	*Piriformospora indica*	**FJ641899**	AF019636	AY505557	**FJ623636**	**FJ623654**
	*Sebacina incrustans*	**FJ641901**	KF000458	**FJ644513**	**FJ623638**	**FJ623653**
	*Tremella mesenterica*	**FJ641904**	AF444433	**FJ644525**	**FJ623641**	**FJ623650**
	*Wallemia sebi*	**FJ641905**	KJ409914	DQ847518	**FJ623642**	**FJ623651**
**Pucciniomycotina**						
	*Agaricostilbum pulcherrimum*	**FJ641896**	GU291274	AJ406402	**FJ623633**	**FJ623646**
	*Microbotryum violaceum*	DQ789983	DQ789984	DQ789982	AET79586	ABG88064
	*Mixia osmundae*	D14163	NR_119614	DQ831009	GAA96338	GAA99857
	*Puccinia graminis*	AY125409	HM131358	AF522177	XP_003334524	XP_003321874
**Ustilaginomycotina**						
	*Exobasidium vaccinii*	**FJ641898**	AB180362	FJ644526	**JF623635**	**FJ623655**
	*Malassezia pachydermis*	DQ457640	DQ411532	AY745724	ABG78261	ABD65910
	*Tilletia goloskokovii*	DQ832247	DQ832248	AY818998	ABH09233	ABH09232
	*Ustilago tritici*	DQ846895	DQ846894	DQ094784	DQ846897	DQ846896
**Entorrhizomycota**						
	*Entorrhiza aschersoniana*	**DQ363318**	**KM359781**	AF009851	**KM359776**	**KM359778**
	*Entorrhiza casparyana*	**FJ641906**	**FJ641906**	AF009852	**FJ623643**	**FJ623652**
	*Entorrhiza parvula*	**KM359779**	**KM359780**	**KM359780**	**KM359775**	**KM359777**
**Earlier diverged fungal lineages**						
**Blastocladiomycota**						
	*Allomyces macrogynus*	EF014364	JN943673	JN941230	EF014385	EF014403, EF014404
	*Catenaria anguillulae*	EF014365	KF700244	FJ804154	EF014386	EF014405
	*Coelomomyces stegomyiae*	AF322406	AY997038	DQ273767	EF014388	EF014406
	*Physoderma maydis*	AY601708	AY997072	DQ273768	DQ294580	DQ302767
**Chytridiomycota**						
	*Synchytrium macrosporum*	DQ322623	AY997095	NG_027565	DQ294605	DQ302792
	*Cladochytrium replicatum*	AY546683	AY997037	AY546688	DQ294587	DQ302774
	*Polychytrium aggregatum*	AY601711	AY997074	AY439068	DQ294584	DQ302770
	*Hyaloraphidium curvatum*	Y17504	AY997055	DQ273771	DQ294585	DQ302772
	*Monoblepharis macrandra*	EF014369	JN882328	AY652933	AF315822	EF014410
	*Boothiomyces macroporosum*	DQ322622	AY997084	DQ273823	DQ294600	DQ302793
**Glomeromycota**						
	*Gigaspora gigantea*	EF014362	AF004684	GQ229222	EF014384	EF014402
	*Funneliformis mosseae*	AY635833	AY997053	DQ273793	EF014383	EF014400, EF014401
**Microsporidia**						
	*Encephalitozoon cuniculi*	L07255	EU001237	AJ005581	NM_001040904	CAD25744
**Neocallimastigomycota**						
	*Neocallimastix frontalis*	EF014370	AY429664	JN939158	EF014394	EF014411, EF014412
**traditional Zygomycota**						
** Entomophthoromycota**						
	*Basidiobolus ranarum*	D29946	AY997030	EF392409	EF014382	EF014399
	*Conidiobolus coronatus*	D29947	AY997041	AY546691	DQ294591	DQ302779
	*Entomophthora muscae*	AY635820	AY997047	DQ273772	DQ294590	DQ302778
**Kickxellomycotina**						
	*Capniomyces stellatus*	AF007531	EF396189	DQ367491	EF014379	EF014396
	*Furculomyces boomerangus*	AF007535	AY997050	DQ273809	EF014380	EF014397
	*Coemansia reversa*	AY546685	AY997039	AY546689	DQ294594	DQ302783
	*Zancudomyces culisetae*	AF007540	AY997089	DQ273773	EF014378	EF014395
**Mucoromycotina**						
	*Mucor hiemalis*	AF113428	KC466522	AF113468	EF014381	EF014398
**Mortierellomycotina**						
	*Mortierella verticillata*	AF157145	AY997063	DQ273794	DQ294595	DQ302784
**Outgroup (Metazoa)**						
	*Caenorhabditis elegans*	AY284652	NR_000057	X03680	NP_500523	Q10578
	*Drosophila melanogaster*	KC177303	M21017	KC177803	AAF48057	AHN57322
	*Homo sapiens*	K03432	NR_003285	NR_003287	NP_000928	NP_000928
	*Mus musculus*	X00686	NR_003280	NR_003279	NP_033115	NP_722493

Accession numbers of sequences generated in this study are indicated by boldface. Voucher numbers can be inferred from the respective GenBank accessions.

We assembled a dataset comprising ribosomal (18S+5.8S+28S) and protein-coding (*rpb*1+*rpb*2) sequences of *Entorrhiza* and 51 samples representing the main clades of the Fungi, see Table 1. Our dataset was complemented with DNA sequences downloaded from GenBank (http://www.ncbi.nlm.nih.gov), mainly from studies by James *et al*. [16] and Liu *et al*. [17]. The final alignment has been deposited in TreeBASE under submission ID S15778.

Genomic DNA was extracted from root galls of *Entorrhiza aschersoniana*, *E*. *casparyana*, and *E*. *parvula* (one gall per species) and from basidiocarps or strains of additional fungi using a DNAeasy Plant Mini Kit (Qiagen, Hilden, Germany) or an InnuPREP Plant DNA Kit (Analytik Jena, Jena, Germany) according to the manufacturers protocols. Galls were washed carefully with tap water and rinsed several times in sterile double-distilled water. All fungal samples were ground in liquid nitrogen, suspended in 400 μl extraction buffer and incubated for 40 min at 65°C. Polymerase chain reaction (PCR) primers RPB1-A and RPB1-C [18] were used to target domains A–C of *rpb*1, and *rpb*2 was amplified with the primer pair RPB2-5F and RPB2-11bR [19]. PCRs were performed using Phusion High-Fidelity DNA Polymerase (Finnzymes Oy, Vantaa, Finland), following the protocol recommended by the manufacturer with annealing temperatures of approximately 5°C above the mean primer melting temperatures, for 30 cycles. Amplicons of positive PCRs were purified using ExoSAP-IT (USB Corporation, Cleveland, OH, USA), and diluted 1:20. Sequencing of *rpb*1 and *rpb*2 genes was carried out using amplification primers and with additional primers as described in Liu *et al*. [17]. Cycle sequencing was accomplished using a 1:6 diluted BigDye Terminator v3.1 Cycle Sequencing Kit (Applied Biosystems, Foster City, CA, USA). Sequencing was performed on an ABI Prism 3130*xl* Genetic Analyzer (Applied Biosystems). Forward and reverse sequence chromatograms were checked for accuracy and edited using Sequencher v. 4.1 (Gene Codes Corporation, Ann Arbor, MI, USA).

Nucleotide sequences of *rpb*1 and *rpb*2 were aligned separately in DIALIGN2-2 [20] with assessments of both nucleotide and amino acid homology (option-*nt*). From aligned datasets, exon C in *rpb*1 and exons 4 and 5 in *rpb*2 were identified via comparisons with annotated coding sequences of *Phragmidium* (EF014377 and AY485630) in Se-Al v. 2.0a11 (http://tree.bio.ed.ac.uk/software/seal). In addition, for the identification of introns we used annotated sequences of *Neolecta vitellina* (EF014375) and *Taphrina deformans* (EF014374) from GenBank, and the GT-AG border motifs [21]. The final *rpb*1 and *rpb*2 alignments had lengths of 134 and 595 amino acids, respectively. Ribosomal DNA sequences were aligned with the MAFFT server (http://mafft.cbrc.jp/alignment/server/index.html) [22] separately for the 18S, 5.8S, and 28S regions, using the E-INS-i option. Ambiguously aligned regions were then removed with Gblocks [23], with gaps allowed at an alignment position when present in more than half of the sequences, a minimum block length of five, a minimum number of sequences for a conserved position or a flanking position of 29, and a maximum number of contiguous nonconserved positions of eight. Alignment length for the trimmed 18S, 5.8S, and 28S datasets were 1596, 155, and 1167 nucleotides, respectively. The single-gene alignments were then concatenated into a single mixed DNA / amino acid dataset of a total of 3,647 characters.

For phylogenetic analysis of this dataset we used maximum likelihood (ML), Bayesian MCMC (BI), and maximum parsimony (MP) based approaches. RAxML v. 8.0.24 [24] was employed in a parallelized version available at the CIPRES portal (http://www.phylo.org), using the GTR model of DNA substitution and starting trees inferred from 1,000 rapid bootstrap analyses [25] in a heuristic search for the tree with the highest likelihood (*-f a* option). For the *rpb*1/*rpb*2 amino acid data, the LG model [26] was chosen using the model selection function of RAxML (AUTO option). To model rate heterogeneity, Gamma-distributed substitution rates were assumed both for the rDNA and the protein alignment parts. RAxML was also employed to infer ML trees for all single-tree alignments (with settings as described above).

Second, we analyzed the datasets using BI as implemented in MrBayes v. 3.2.2 [27]. For each dataset two parallel runs were performed, each over 5 million generations, with a sample frequency of 100 and a burn-in percentage of 20%, integrating the stored trees from the two parallel runs in a single majority-rule consensus. For the rDNA parts we used the GTR model, and for the amino acid parts we allowed sampling of the available amino acid substitution models during the MCMC process (*aamodelpr = mixed*); rate heterogeneity was modelled with unlinked Gamma distributions. We also unlinked all substitution models, state frequencies, rate multipliers, and branch lengths for the partition subsets.

Third, we did MP bootstrap analyses in PAUP* v. 4a136 [28] with 1000 bootstrap replicates. In each bootstrap replicate we performed ten heuristic searches, with starting trees obtained by subsequently adding sequences in random order and using TBR branch swapping (*multrees* option switched on and *steepest descent* option switched off). Gaps were treated as missing data.

We also did an ML analysis in RAxML to search for the best tree with the constraint that the union of Basidiomycota and *Entorrhiza* is monophyletic (with additional settings as described above). A Shimodaira-Hasegawa (SH) test [29] was performed to test whether this tree was significantly worse than the overall best tree.

Finally, we calculated internode certainty (ICA) and tree certainty (TCA) values for the multigene and the single-gene ML trees [30]. ICA is a measure of the amount of information that relates to a branch and is calculated from the frequency distribution of the relevant bipartitions for this branch in the bootstrap trees, i.e., from the frequency of the bipartition represented by this branch (which equals the bootstrap value) and the frequencies of those bipartitions present in the bootstrap trees that are in conflict with this branch [30]. TCA is the average of all ICA values in a tree and facilitates the comparison of different trees with respect to their mean internode certainty.

### Microscopy

For teliospore germination the following specimen was used: *Entorrhiza casparyana* on *Juncus articulatus*, Germany, Baden-Württemberg, Triensbach, Reusenberg, 49°09'10.3"N 9°59'48.8"E, 10 Oct 1988, leg. R. Bauer 942. Galls were placed in water in petri dishes, incubated at 7°C and examined weekly.

For light microscopy (LM), the following specimens were used: *Entorrhiza casparyana* on *Juncus articulatus*, Switzerland, near Preda, Albuda pass, 46°35'03.5"N 9°51'11.9"E, 1976, leg. G. Deml and *E*. *casparyana* on *J*. *articulatus* R. Bauer 942 (see above). Living material was examined with an Axioplan microscope (Zeiss, Oberkochen, Germany) using bright field, phase contrast, or Nomarski interference contrast optics. For nuclear staining, material was dried on slides for 10 min at room temperature, then fixed with a 3:1 mixture of 92% ethanol and acetic acid for 30 min. After rinsing the material several times with water, it was hydrolysed in 60°C HCl (1N) for 7 min, rinsed again in one change of water and five changes of phosphate buffer (pH 7), then stained for 2 h in Giemsa stock solution and phosphate buffer (1:9, v/v). It was then rinsed once more in phosphate buffer, dipped in water, and mounted with a cover glass.

For transmission electron microscopy (TEM) *Entorrhiza casparyana* on *Juncus articulatus* R. Bauer 942 was used (see above). Galls were fixed with 2% glutaraldehyde in 0.1 M sodium cacodylate buffer (pH 7.2) at room temperature. After six transfers in 0.1 M sodium cacodylate buffer the samples were post-fixed in 1% osmium tetroxide in the same buffer for 1 h in the dark. The samples were washed in distilled water and then stained in 1% aqueous uranyl acetate for 1 h in the dark. After five washes in distilled water, the samples were dehydrated in a graded series of acetone, using 10 min-changes at 25%, 50%, 70%, 95%, and three times at 100%. Samples were embedded in Spurr’s plastic and then cut with a diamond knife-equipped Reichert-Jung Ultracut E microtome and collected on formvar-coated slot grids. They were post-stained with uranyl acetate and lead citrate at room temperature for 5 min, and then washed with distilled water. Samples were examined with a Zeiss transmission electron microscope operating at 80 kV.

For scanning electron microscopy (SEM), the following specimen was used: *Entorrhiza casparyana* on *Juncus articulatus*, Russia, Karelia, Kiwatsch, 62°12'02.2"N 34°16'07.4"E, August 2010, leg. D. Begerow 1234. A gall was fixed in 5% formalin – 5% acetic acid – 90% alcohol *v/v* (FAA), washed with deionised water (3–10 min), dehydrated in an ascending ethanol series, transferred to formaldehyde dimethyl acetal (FDA) for 24 h [31], critical-point dried, split with a razor blade in two halves and sputter-coated with gold for 220 s. The material was examined with a DSM 950 (Zeiss) at 15 kV and the results were documented using Digital Image Processing Software v. 2.2 (DIPS, Leipzig, Germany).

### Nomenclature

The electronic version of this article in Portable Document Format (PDF) in a work with an ISSN or ISBN will represent a published work according to the International Code of Nomenclature for algae, fungi, and plants, and hence the new names contained in the electronic publication of a PLoS ONE article are effectively published under that Code from the electronic edition alone, so there is no longer any need to provide printed copies. In addition, the taxon species name contained in this work was submitted to MycoBank, from where they will be made available to the Global Names Index. The MycoBank number can be resolved and the associated information viewed through any standard web browser by appending the MycoBank number contained in this publication to the prefix www.mycobank.org/MB. The online version of this work is archived and available from the following digital repositories: PubMed Central, LOCKSS.

## Results

### Molecular phylogenetic analysis

**Fig 2:**
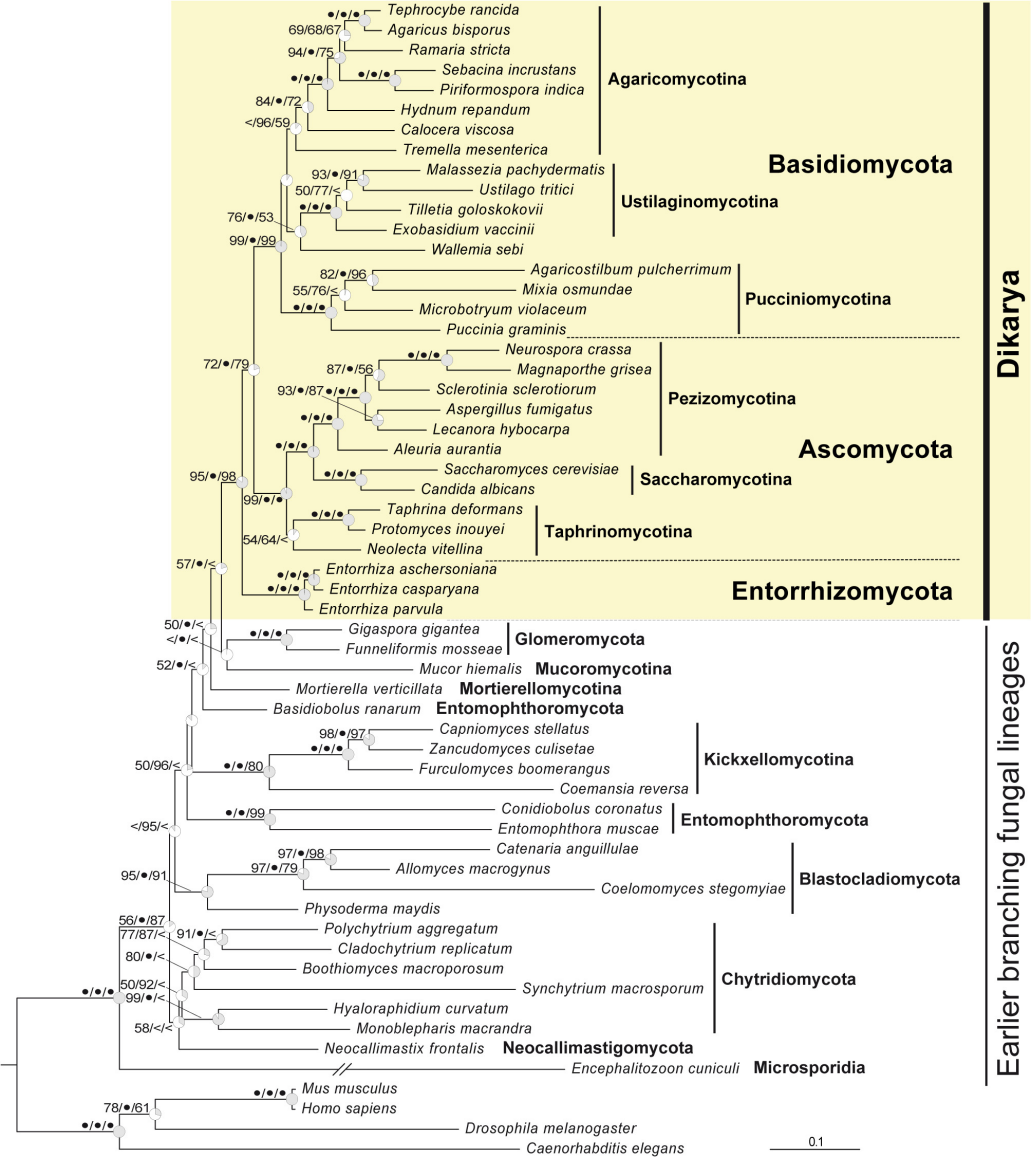
Maximum likelihood tree showing the phylogenetic position of the genus *Entorrhiza* within the kingdom Fungi derived from a combined dataset of rDNA (18S, 5.8S and 28S) and amino acid (RPB1 and RPB2) sequences. Branch support is given as maximum likelihood bootstrap percentage (1,000 replicates) / Bayesian posterior probability (consensus of two independent MCMC processes, each with four chains over five million generations) / maximum parsimony bootstrap (1,000 replicates). Values of 100% are designated with bullets, values below 50% are omitted or designated by <. Grey sectors in the clock symbols illustrate internode confidence values (ICA [30]) of the supporting branch; counterclockwise orientation designates a negative ICA value, i.e., an instance where a conflicting bipartition occurs more frequently in the bootstrap trees than the bipartition defined by this branch. The tree was rooted with the Metazoa branch. The *Encephalitozoon* branch was scaled with a factor of 0.5 for graphical reasons. For GenBank accession numbers see Table 1.

The phylogenetic position of the genus *Entorrhiza* derived from a combined dataset of rDNA (18S, 5.8S and 28S) and amino acid (RPB1 and RPB2) sequences is shown in Fig 2. *Entorrhiza aschersoniana*, *E*. *casparyana* and *E*. *parvula* formed a highly supported monophyletic group in all phylogenetic analyses. In the ML analysis this group appeared as the most basal group of the Dikarya, a position which received 72% bootstrap support in ML and 79% in MP analysis, and a BI support of 100%. However, the branch supporting the monophyletic union of Ascomycota and Basidiomycota (without *Entorrhiza*) received an ICA value as low as 0.197, which indicates a considerable frequency of conflicting bipartitions in the set of the ML bootstrap trees. In the best tree found in the ML analysis under the constraint that Basidiomycota plus *Entorrhiza* is monophyletic, *Entorrhiza* was the sister group to the Basidiomycota (not shown). In the SH test this tree was not significantly worse than the overall best tree found (shown in Fig 2) at a significance level of *p* = 0.05.

TCA values for the single-gene ML trees were 0.37 (18S), 0.34 (28S), 0.01 (5.8S), 0.24 (*rpb*1), and 0.49 (*rpb*2). In the ML tree for the gene with the highest TCA value (*rpb*2) *Entorrhiza* appeared as the most basal group in the Dikarya, and the union of Basidiomycota (without *Entorrhiza*) and Ascomycota received an ICA value of 0.534 (not shown).

Backbone relationships of the earlier diverged fungal lineages were poorly resolved, as indicated by low bootstrap values and low or even negative ICA values (Fig 2).

### Morphology

**Fig 3:**
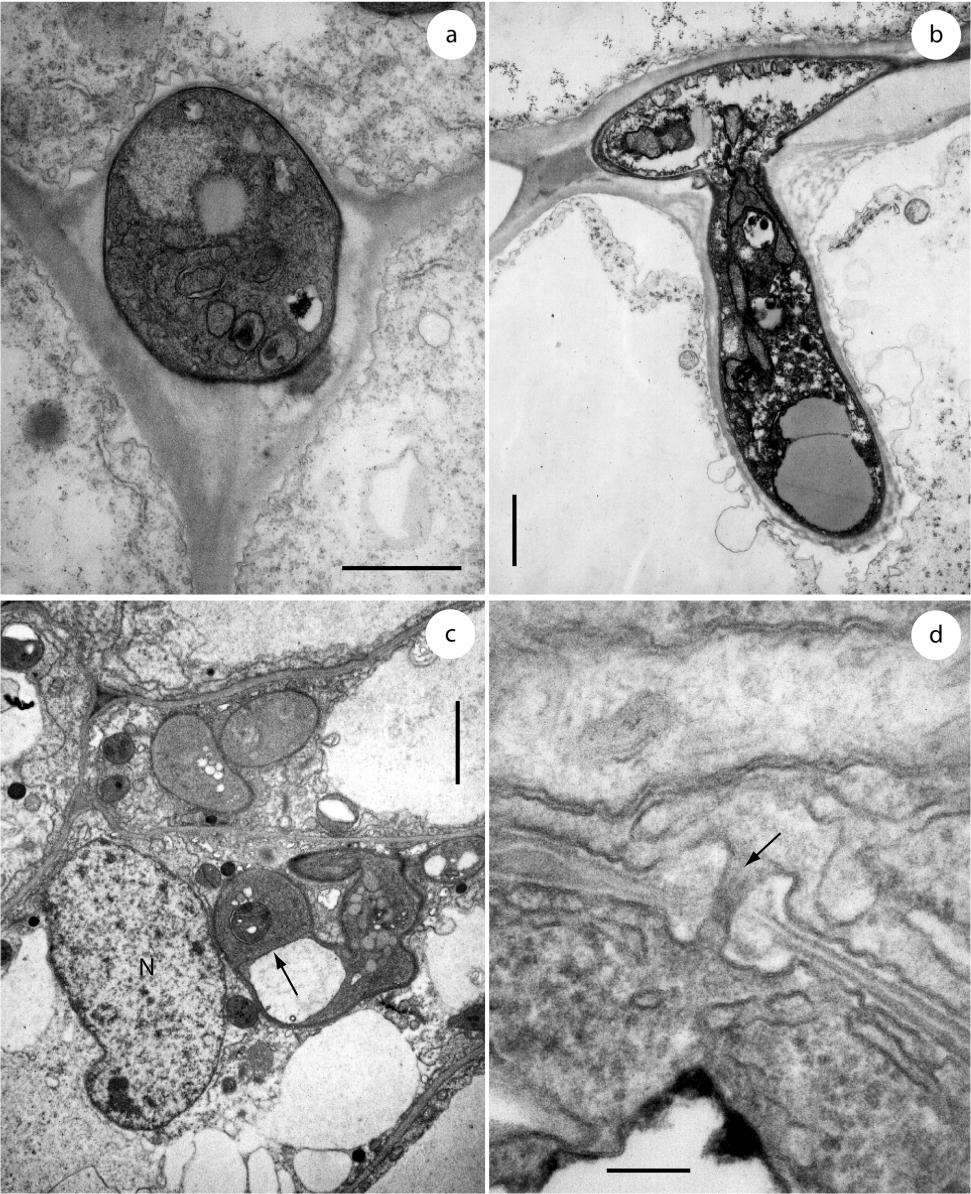
Soral hyphae of *Entorrhiza casparyana* in root galls of *Juncus articulatus*, as seen by TEM. **(a)** Section through an intercellular hypha surrounded by plant cells. **(b)** Section through an intercellular hypha extended into a cortical cell. Note that the intracellular part of the hypha is surrounded by the host plasma membrane. **(c)** Section through two hyphal coils in neighbouring cortical cells of *J*. *articulatus*. One host nucleus is visible at N, and a hyphal septum is visible at the arrow. **(d)** Section through a hyphal septum showing a dolipore without membranous caps (arrow). Note the basidiomycetous tripartite profile of the septum wall. Scale bar is 1 μm in (a-b), 2 μm in (c), and 0.1 μm in (d).

**Fig 4:**
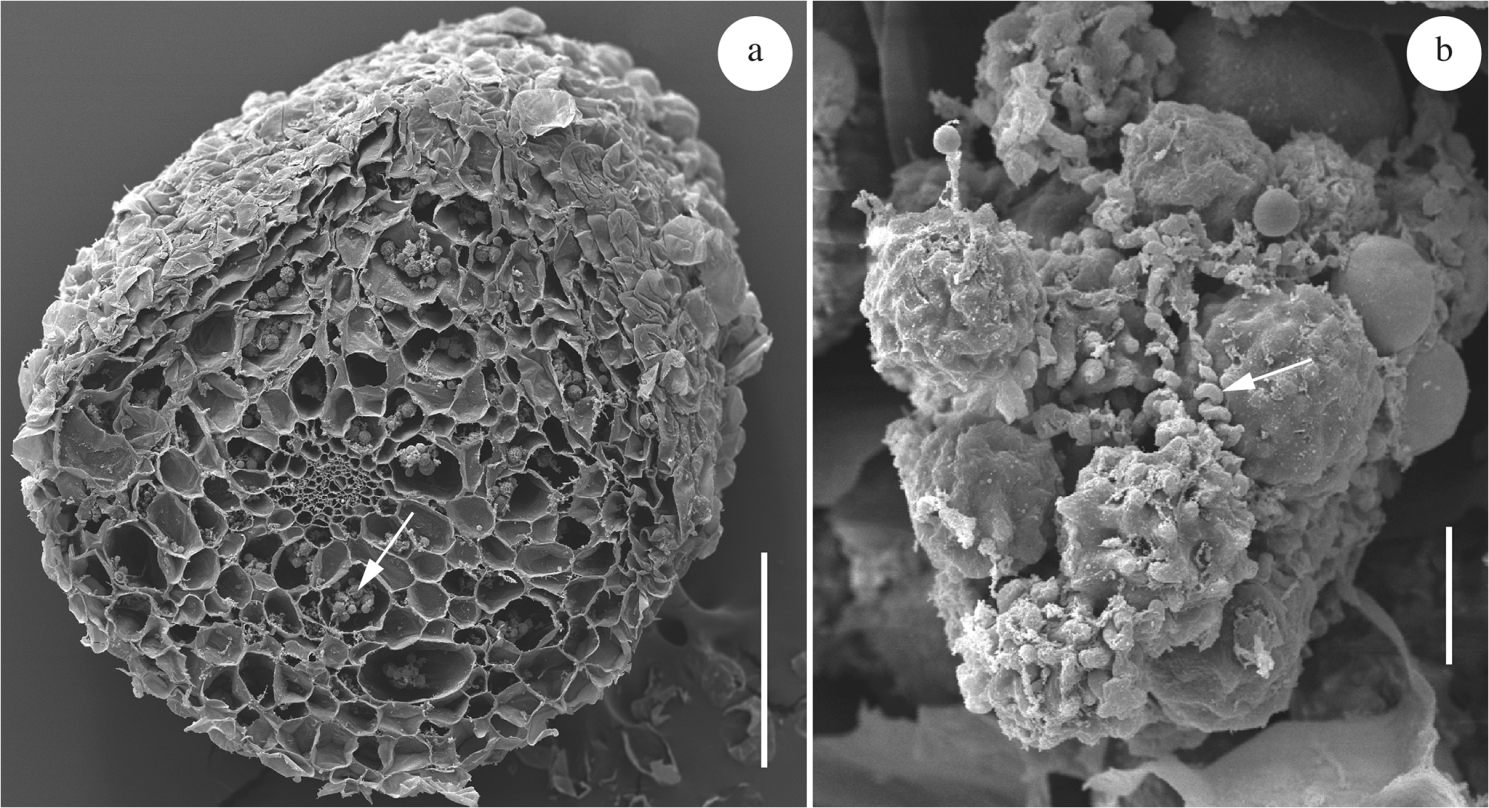
Sections through galls of *Entorrhiza casparyana* on *Juncus articulatus*, as seen by SEM. **(a)** Section showing intracellular teliospore packets (one is indicated by an arrow). **(b)** Teliospore packet in detail, showing teliospores and hyphal spirals (one is indicated by an arrow). Scale bar is 1 mm in (a) and 20 μm in (b).

**Fig 5:**
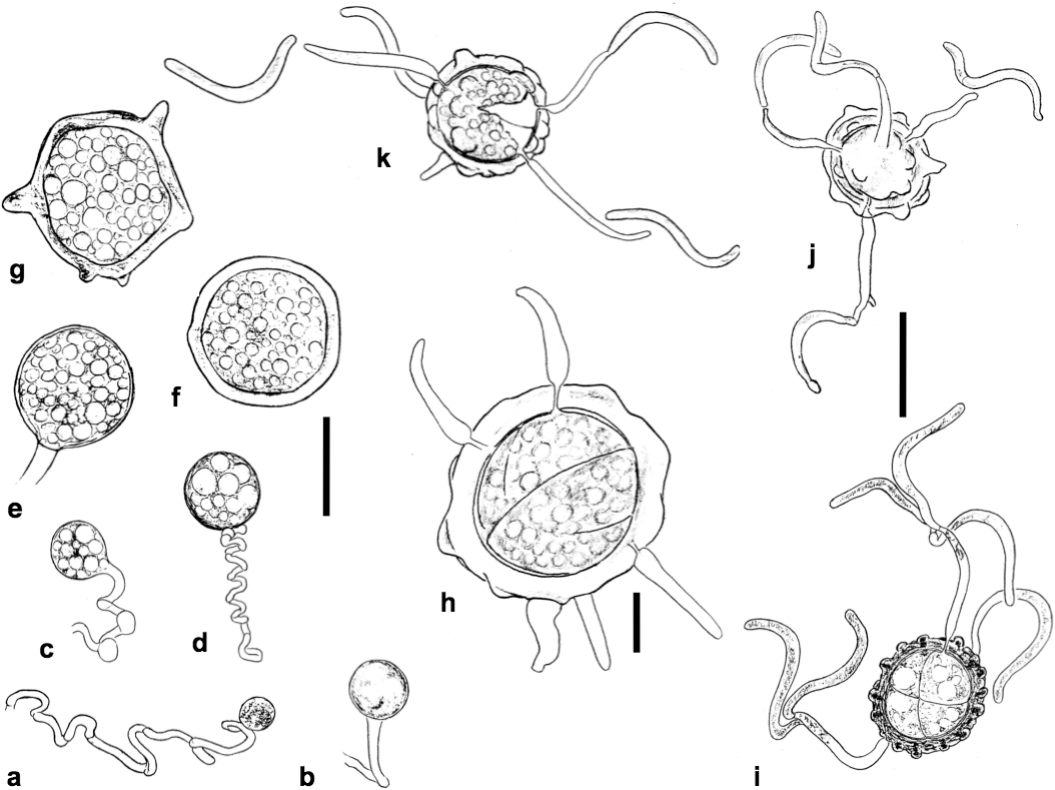
Line drawings of *Entorrhiza casparyana*. **(a-g)** Intracellular more or less coiled hyphae and teliospores in different developing stages (host cells not drawn). **(h-k)** Germinating teliospores in different developing stages. Note the sigmoid shape of the propagules. Scale bar is 20 μm in (a-g, j-k) and 10 μm in (h).

*Entorrhiza casparyana* caused galls on roots of *Juncus articulatus* (Fig 1). Our morphological analyses allowed a reconstruction of the infection process as follows: Hyphae first grew intercellularly between cortical cells (Fig 3A), and later gave rise to intracellular hyphae (Fig 3B). Intracellular hyphae were sparsely constricted at the penetration point (Fig 3B) and differed morphologically from the intercellular hyphae. They were generally coiled and terminated in the host cells with the formation of teliospores (Figs 3C and 4A and 4B and 5A–5G). Intercellular as well as intracellular hyphae were regularly septate and possessed dolipores without bands or caps (Figs 3C and 3D and 5A–5D). Clamp connections were absent. Intercellular as well as intracellular hyphae had electron-opaque, fibrillate walls. Septa showed a tripartite profile (Fig 3D).

**Fig 6:**
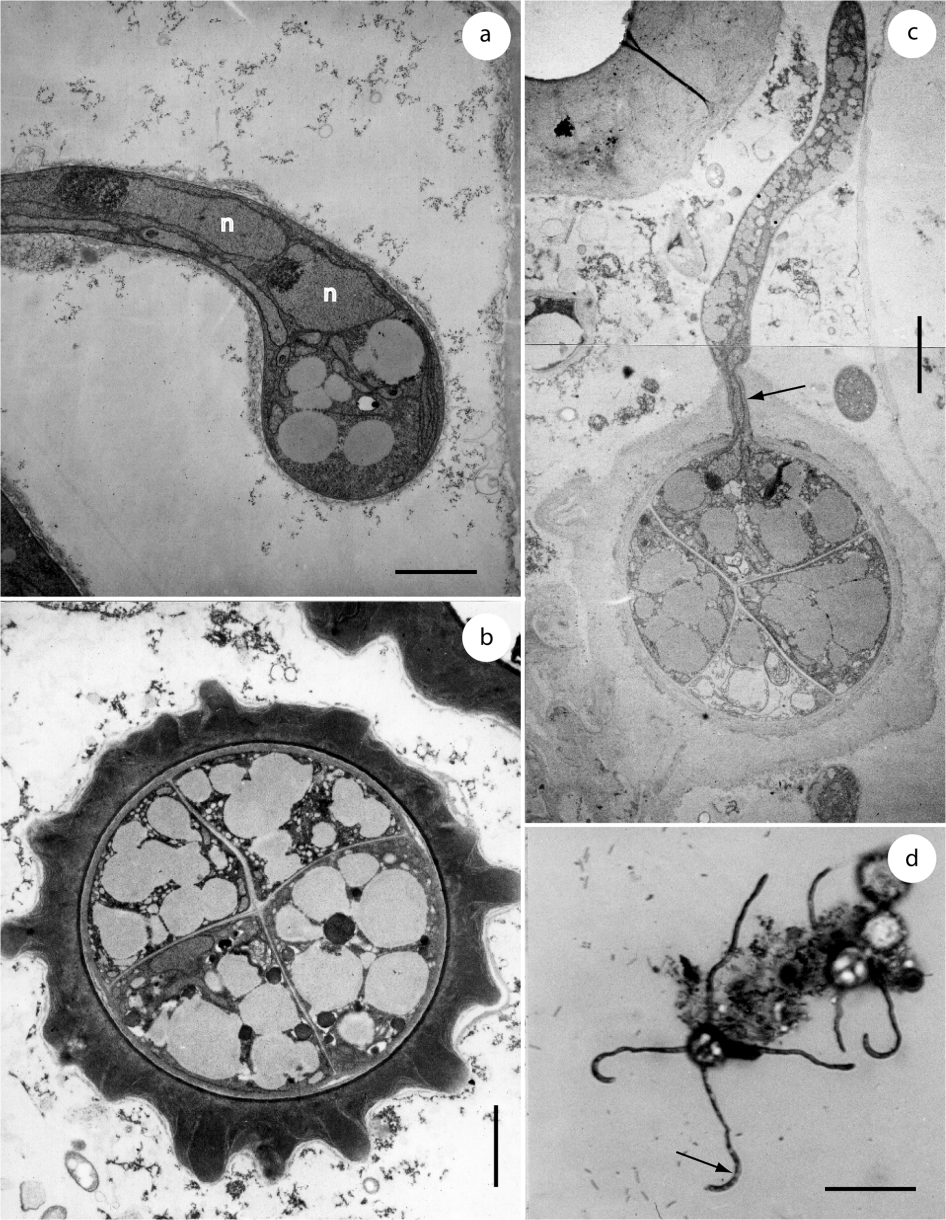
Teliospore formation and germination of *Entorrhiza casparyana*, as seen by TEM (a-c) or LM (d). **(a)** Section through an intracellular hyphal coil showing a terminal teliospore initial with two nuclei (n). **(b)** Section through a teliospore tetrad showing the cruciform septation. **(c)** Section through a teliospore tetrad showing the germination of one of the four teliospore compartments. One nucleus is visible at the arrow, probably migrating into the germination hypha. **(d)** Light microscopic micrograph of a germinating teliospore illustrated to show the synchronous development of the germination hyphae and the terminal sigmoid propagules (one is indicated by an arrow). Scale bar is 2 μm in (a-b), 3 μm in (c), and 40 μm in (d).

Because of the coiled nature of the intracellular hyphae they were difficult to trace in the TEM analyses. By examining serial sections we mostly detected two nuclei per hyphal cell (Fig 6A), but occasionally up to four nuclei could be observed in one cell. In young teliospores we mostly observed two nuclei. In Giemsa-stained material we usually observed one large nucleus in older teliospores that were stored in water at 7°C for two months.

Germination in *Entorrhiza casparyana* started after teliospores had been stored in water at 7°C for 76 days. First, they became regularly two-celled by septation. Subsequently, the two segments in each primary teliospore divided synchronously so that regularly cruciform teliospore tetrads could be observed (Figs 5H–5K and 6B and 6C). The internal septa also had dolipores. In serial sections, we detected only one nucleus per teliospore compartment (Fig 6C). Germination hyphae arose synchronously from each compartment (Fig 6C and 6D). Finally, sigmoid monokaryotic propagules developed on each germination hypha (Figs 5H–5K and 6D). In our germination experiments on agar media these propagules did not germinate.

**Fig 7:**
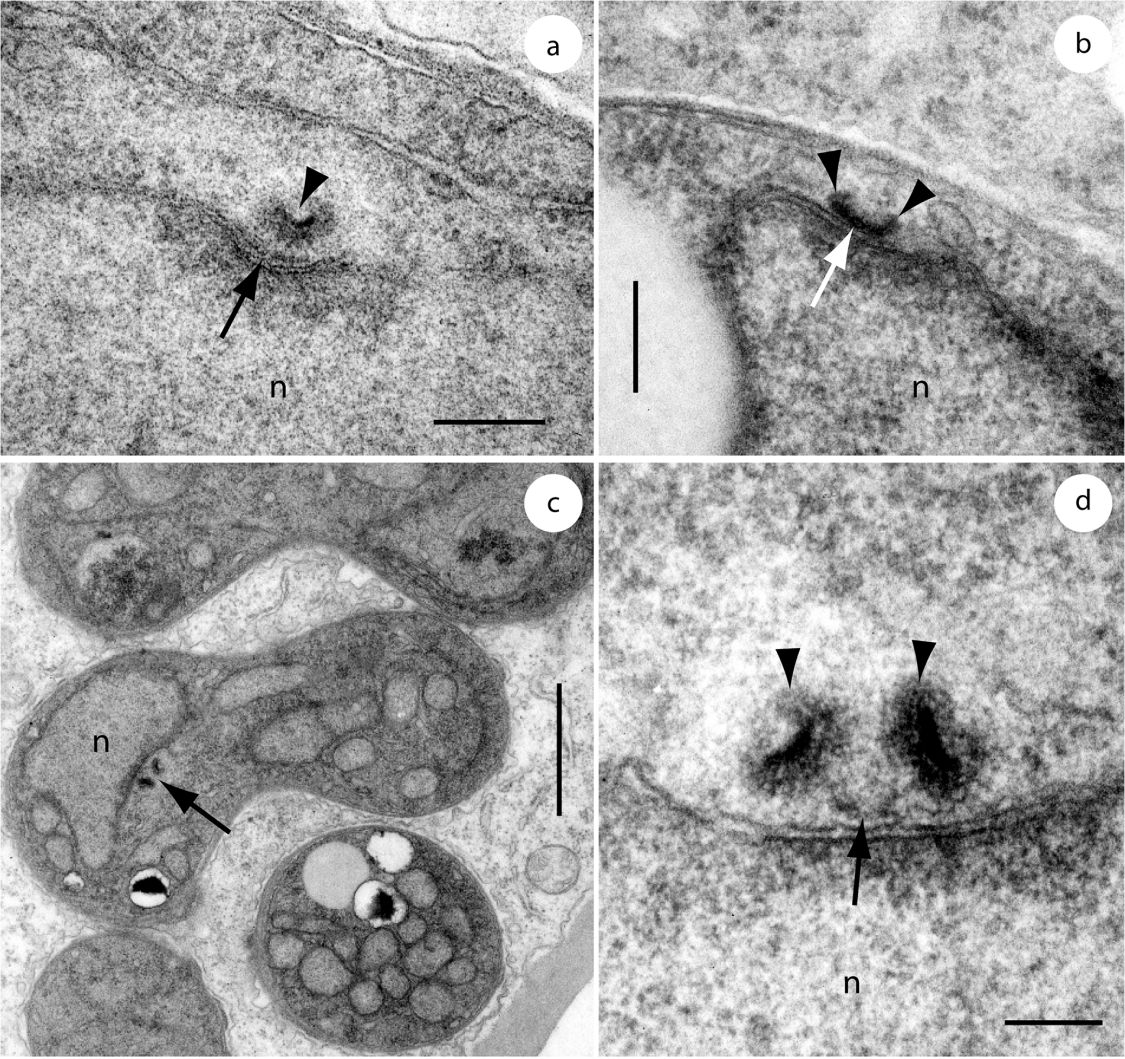
Spindle pole bodies (SPBs) of *Entorrhiza casparyana*, as seen by TEM. **(a)** Median section through a postmitotic / early interphasic SPB showing the original hemispherical SPB with an internal electron-opaque layer (arrowhead) and an additional bar (arrowhead) next to the nuclear envelope. **(b)** Longitudinal section through a mid-interphasic SPB. The double structure of the SPB has begun to reconstitute by growth of SPB elements (arrowheads) at the ends of the electron-opaque bar (arrow). **(c)** Section through an intracellular hyphal coil showing one nucleus with a longitudinally sectioned premitotic SPB (arrow). **(d)** Detail of (c) illustrated to show the two enlarged SPB elements (arrowheads) connected by an elongated middle piece (arrow). Note the internal layering of the SPB elements. n, nucleus. Scale bar is 0.2 μm in (a-b), 1 μm in (c), and 0.1 μm in (d).

The interphasic mitotic spindle pole body (SPB) in *E*. *casparyana* is a single extranuclear hemispherical body that in transition from telophase to interphase contained a convex and completely internalized electron-opaque layer. SPB replication began with the appearance of an electron-dense bar on the nuclear periphery of the original SPB next to the nucleus (Fig 7A). Next, the original SPB disappeared and the bar transformed into a double-structured SPB. SPB material formed at each end of the bar and developed into the two SPB elements of the double-structured SPB (Fig 7B). Then the SPB elements increased in size and the layering became evident, whereas the middle piece decreased in size and finally disappeared, so that in late interphase / early prophase the two SPB elements appeared separated from each other (Fig 7C and 7D).

### Taxonomy

Our study indicates that the *Entorrhiza* lineage does not belong to any of the described phyla of the Fungi (for the taxa see [14], [32–33]). Accordingly, we propose the following new phylum:

**Entorrhizomycota R. Bauer, Garnica, Oberw., K. Riess, M. Weiß & Begerow, *phylum nov*.** (Figs 1–7)

[MycoBank no: MB808783]

Members of the Fungi sensu Hibbett *et al*. [14] infecting roots with regularly septate coiled hyphae. Septal pores without Woronin bodies or membrane caps.

Remarks: Only the genus *Entorrhiza* C.A. Weber [1: p. 378] is included. Entorrhizomycetes was proposed by Begerow *et al*. [12: p. 908]; Entorrhizomycetidae, Entorrhizales, and Entorrhizaceae were established by Bauer & Oberwinkler in Bauer *et al*. [7: p. 1311].

## Discussion

### Phylogenetic placement of *Entorrhiza*

So far, only seven rDNA sequences have been published for four of 14 known species of *Entorrhiza*. In the analyses of Begerow *et al*. [12], [15] the taxon sampling was restricted to the subphylum Basidiomycota. However, these authors already noted that in analyses with several ascomycetes and zygomycetes “*Entorrhiza* was not always included in the Ustilaginomycetes” [15]. Matheny *et al*. [13] suggested that *Entorrhiza* is not nested within any described subphylum and might also represent the sister group of the rest of the Basidiomycota (see also [14]).

In general, these studies indicated that phylogenetic analyses based on rDNA sequences alone apparently do not allow the derivation of a statistically robust phylogenetic hypothesis for *Entorrhiza*. Therefore, we additionally sequenced the nuclear protein-coding genes *rpb*1 and *rpb*2 for three *Entorrhiza* species associated with different host plant families, *E*. *aschersoniana*, *E*. *casparyana*, and *E*. *parvula*. Because the spores of *Entorrhiza* develop intracellularly and are therefore not separable from the host tissue, and because the propagules of *E*. *casparyana* did not germinate in our germination experiments and we therefore did not obtain a culture, it has been impossible to sequence more genes to date.

Our combined 5-gene sequence analysis (Fig 2) is consistent with previous multi-gene studies [16–17]. In particular, Basidiomycota, Ascomycota, Glomeromycota, Kickxellomycotina, and Blastocladiomycota appear with high support, whereas Chytridiomycota was only moderately supported. Microsporidia as sister taxon to Fungi and a sister-group relationship of Ascomycota and Basidiomycota are also in agreement with the results of previous studies in most aspects [16–17]. In addition, and in contrast with the results of Matheny *et al*. [13], our analysis yields a supported hypothesis for the phylogenetic position of *Entorrhiza*, suggesting that the *Entorrhiza* lineage is a monophyletic sister taxon to the rest of the Dikarya. However, branch support for the non-*Entorrhiza* Dikarya was only moderate in our five-gene ML (79%) and MP (72%) analyses (Fig 2). Only in Bayesian MCMC analysis did this group receive a branch support of 100% estimated posterior probability. We also found that the monophyletic non-*Entorrhiza* Dikarya received a low internode certainty value (ICA) of only 0.197 in the ML bootstrap analysis (Fig 2), indicating that at least one conflicting grouping is present in a considerable number of bootstrap replicates [29].

Thus, we also tested the alternative hypothesis that *Entorrhiza* is included in the Basidiomycota. Under this constraint, *Entorrhiza* appeared as the most basal lineage in the Basidiomycota in ML analysis (not shown), which taxonomically would be consistent with a new phylum Entorrhizomycota as well. In an SH test this constraint tree was not significantly worse than the overall ML tree shown in Fig 2.

On the whole, our five-gene analyses suggest that *Entorrhiza* is the sister group of all other Dikarya, though we cannot significantly reject an alternative topology in which *Entorrhiza* is sister to Basidiomycota. Both alternatives are consistent with our proposal of a new phylum Entorrhizomycota. Additional evidence for the first scenario comes from a closer inspection of the single-gene analyses. We found the highest average internode support (TCA value) in the ML tree inferred from the *rpb*2 alignment, indicating that bootstrap consistency in this analysis is higher than those in the ML analyses of the other genes analyzed here [34]. According to the *rpb*2 ML tree *Entorrhiza* is sister to the rest of the Dikarya. Future phylogenomic analyses hopefully provide a final confirmation or falsification of this hypothesis, once genomes of *Entorrhiza* have become available.

### Subcellular and cellular characters

Morphologically, *Entorrhiza* shares more traits with Basidiomycota than with Ascomycota. As in basidiomycetes, but in contrast to ascomycetes, the vegetative hyphal cells are dikaryotic, the hyphal walls have an electron-opaque fibrillate substructure and the septa show a tripartite profile in *E*. *casparyana* [35–36]. In addition, SPB form, structure, and behavior in *Entorrhiza casparyana* are basidiomycetous (see [37] and the references therein). In the transition from telophase to interphase, the SPB is a single extranuclear hemispherical body that contains a completely internalised electron-opaque layer that is convex relative to the nuclear surface. SPB replication in *Entorrhiza* occurs as it does in basidiomycetes in general. An electron-dense bar first appears on the nuclear periphery of the original SPB next to the nucleus. After that the original SPB disappears and the bar transforms into a double-structured SPB. SPB material forms at each end of the bar, which develops into the two SPB elements of the double-structured SPB. Subsequently, the SPB elements increase in size and the layering becomes evident (Fig 7), whereas the middle piece decreases in size and finally disappears so that in late interphase / early prophase the two SPB elements are separated from each other. In contrast with *Entorrhiza casparyana* and basidiomycetes, the SPBs of ascomycetes are discoidal in form and duplicate by fission [38].

Teliospores of *Entorrhiza* germinate internally by becoming four-celled with cruciform septation [1], [8–9]. Although the meiotic stages have not been studied in detail, the changes in numbers of nuclei from binucleate to mononucleate to four-nucleate [8–9], the synchronous second division of the two teliospore segments, and the synchronous germination of the resulting teliospore tetrad indicate that they represent meiosporangia. Interestingly, based on experimental studies on *Gymnosporangium*, *Helicogloea* and *Platygloea*, Bauer and Oberwinkler [39] demonstrated that the basidial cells in phragmobasidia essentially represent meiospores and that the meiosporangial dispersal propagules accordingly represent secondary vegetative cells, comparable to the yeasts of *Ustilago*, or ballistosporic conidia of the rusts [40] (see also [41]). As particularly indicated by the formation of septal pores, the septation of the *Entorrhiza* teliospores is a hyphal septation, which is essentially identical to that of external transversely-septate phragmobasidia (see [42], and the references therein). On the other hand, the meiosporangial tetrads in *Entorrhiza* are also essentially identical to those of *Tetragoniomyces uliginosus*, a tremelloid mycoparasite [43]. Interestingly, *T*. *uliginosus* occurs on decaying plant material in wet habitats. As already noted by Oberwinkler and Bandoni [43] for *T*. *uliginosus*, the meiosporangial tetrads of *Entorrhiza* may also be an adaption for water dispersal in the soil. In addition, the basidiospores in *Entorrhiza* resemble the conidia of aquatic hyphomycetes [44]. Thus, a plausible scenario for the evolution of the aerial dispersal of meiospores in the Dikarya may, as a first step, have involved the formation of external elongate transversely-septate phragmobasidia in combination with the ballistosporic mechanism, with subsequent repeated evolution of holobasidia that apically give rise to ballistospores, allowing a denser packing of the basidia in hymenia and a more effective aerial spore release.

The meiosporangial tetrads in *Entorrhiza* also resemble the meiospore tetrads in asci of early diverged lineages of ascomycetes, e.g., in *Saccharomyces* and *Schizosaccharomyces* [45–46]. At first glance, the septation within phragmobasida and the formation of ascospores look quite different in comparison, but the underlying processes may be homologous [8], [45–46]. To build cell walls, in both processes vesicles accumulate adjacent to an initial membranous body and then fuse with the body and extrude their contents so that the initial body becomes enlarged. The main difference in the two processes is the initial body: in hyphal septation it is the plasma membrane sac, whereas in the development of the ascospore wall it is an ER cisterna that is associated with the cytoplasmic face of the SPB. However, three-dimensional imaging has shown that the plasma membrane sac and the ER cisternae are continuous anyway [46].

As for the evolutionary trend to aerial dispersal outlined above for basidiomycetes, from internal meiosporangia via phragmobasidia to holobasidia, we may also postulate an evolutionary trend to aerial dispersal in ascomycetes, via the formation of eutunicate asci.

In summary, meiosporangial tetrads, as in *Entorrhiza*, may represent the dikaryan meiosporangial prototype.

### Coevolution of *Entorrhiza* with land plants

The species of *Entorrhiza* infest members of Cyperaceae and Juncaceae worldwide [2], [5]. The origin of the Dikarya (and thus also of the *Entorrhiza* lineage as its assumed sister group) was roughly dated at around 600 million years BP, more or less parallel to the first appearance of primitive land plants [47–48], whereas the origins of the known extant host groups of the *Entorrhiza* clade, the core Poales, has been dated at around 100 million years BP [49–50]. Because of this discrepancy, we hypothesise that the known species of the *Entorrhiza* clade and their respective host spectra reflect only the tip of the iceberg of a much broader group, comprising perhaps also the lower land plants. Because of the lack of any aboveground signal of infection, the host plants are difficult to detect in nature. There also might be members of the *Entorrhiza* clade that do not cause galls on the roots. To test this hypothesis, we are currently developing specific primers for an efficient molecular detection of this fascinating group of root-colonizing fungi.

### Conclusions

For the phylogenetic placement of *Entorrhiza* two alternative hypotheses are most plausible: (i) *Entorrhiza* is the sister group to the rest of the Dikarya, or (ii) *Entorrhiza* is the sister group to Basidiomycota. The first topology was inferred in our phylogenetic analyses of a five-gene dataset. It is consistent with the interpretation of the *Entorrhiza* meiosporangium as representing the ancestral meiosporangium of Asco- and Basidiomycota. The second scenario, which is in agreement with ultrastructural similarities, could not significantly be rejected in ML analysis of our five-gene dataset. Based on its peculiar combination of morphological features we therefore propose, a new phylum of root-colonizing fungi, the Entorrhizomycota, which is compatible with both alternative phylogenetic positions discussed in this work.
